# Internet-Based Self-Management Support After High-Altitude Climate Treatment for Severe Asthma: Randomized Controlled Trial

**DOI:** 10.2196/13145

**Published:** 2020-07-22

**Authors:** Thijs Beerthuizen, Lucia H Rijssenbeek-Nouwens, Sophia M van Koppen, Rishi J Khusial, Jiska B Snoeck-Stroband, Jacob K Sont

**Affiliations:** 1 Department of Biomedical Data Sciences Section Medical Decision Making Leiden University Medical Center Leiden Netherlands; 2 Dutch Asthma Centre Davos Davos Switzerland

**Keywords:** self-management, pulmonary rehabilitation, severe asthma, quality of life, asthma control, internet, monitoring

## Abstract

**Background:**

In patients with severe asthma, high-altitude climate treatment has been shown to improve asthma control. However, asthma symptoms and limitations may increase after finishing inpatient rehabilitation programs and returning to sea level.

**Objective:**

We assessed the effectiveness of a patient-tailored, internet-based, self-management strategy in addition to usual care after finishing high-altitude climate treatment.

**Methods:**

We performed a randomized controlled trial with a 1-year follow-up in patients from a high-altitude asthma center in Davos, Switzerland. At the end of a 12-week multidisciplinary rehabilitation program, 62 adults with asthma were randomized to receive either internet-based self-management support in addition to usual care (n=33) or usual care only after discharge (n=29). The endpoints were changes in asthma-related quality of life according to the Asthma Quality of Life Questionnaire (AQLQ) (a higher score is better) and asthma control according to the Asthma Control Questionnaire (ACQ) (a lower score is better), with a minimally important difference of 0.5 points for both.

**Results:**

Asthma-related quality of life and asthma control declined over time in the usual care strategy group, whereas there was a slower decline in the internet-based strategy group. For both endpoints, mixed-model analysis showed a significant positive effect in favor of internet-based self-management during follow-up (mean AQLQ score difference 0.39, 95% CI 0.092-0.69; *P*=.01 and ACQ score difference −0.50, 95% CI −0.86 to −0.15; *P*=.006), which was prominent among patients with uncontrolled asthma at discharge (AQLQ score difference 0.59, 95% CI 0.19-0.99; *P*=.003 and ACQ score difference −0.73, 95% CI −1.18 to −0.28; *P*=.002).

**Conclusions:**

Internet-based self-management support was associated with a smaller decline in quality of life and asthma control as compared with usual care, especially in patients with lower asthma control, after completion of high-altitude climate treatment. Internet-based self-management support in adults with severe asthma seems feasible and effective to maintain quality of life and asthma control.

**Trial Registration:**

The trial is registered in the Netherlands Trial Register (NTR1995).

## Introduction

In patients with severe refractory asthma, standard treatment according to international guidelines [1] is often not sufficient for achieving adequate asthma control [2]. Patients with persistent uncontrolled asthma can be referred to specialized asthma high-altitude clinics [3,4]. The population referred for in-patient pulmonary rehabilitation comprises patients with complex somatic and psychosocial problems [5]. The effectiveness of pulmonary in-patient rehabilitation in severe asthma has been established in a previous study that reported relevant improvements in asthma control, quality of life, and clinical outcomes with a 12-week multidisciplinary treatment program involving environmental trigger avoidance in an alpine climate among patients with severe refractory asthma [6]. Pulmonary rehabilitation should be part of an integrated care process and include self-management support. Changing patient behavior and ensuring maintenance are complex processes and require time. Currently, most self-management programs take between 6 and 12 weeks, and a longer duration is associated with greater improvements in physical and psychological functioning [6]. At present, there are sparse data on whether the benefits are sustained beyond the completion of high-altitude climate treatment, and patients are not always amenable to optimal self-management on returning to sea level in their own environment. A recent prospective uncontrolled observational cohort study suggested reduced exacerbation and sustained improvements in asthma control 12 months after high-altitude climate treatment for severe asthma as compared to the findings before admission, with only a slight relapse after discharge [7,8]. To sustain these improvements in long-term outcomes, appropriate self-management behavior during and after discharge is considered essential [9]. In order to achieve a sustained optimal long-term quality of life, we need a dependable system of coordinated health care intervention and communication, and components that include self-management support. Innovative forms of self-management support, including an online community, monitoring, communication, an action plan, and motivational feedback via the internet, have high potential to improve long-term outcomes.

Several recent studies have shown the feasibility and effectiveness of telemonitoring and self-management support [10-14]. Earlier studies of our group have demonstrated that internet-based self-management support improves quality of life, the number of symptom-free days, and clinical outcomes [15,16] in patients with partly controlled asthma.

However, the long-term effectiveness of sustaining self-management support via the internet in patients with severe asthma, who have completed in-patient pulmonary rehabilitation, has not been determined yet.

We hypothesize that self-management support via an internet-based service in addition to usual care will improve health-related quality of life and asthma control in patients with severe asthma who have completed pulmonary rehabilitation in a specialized high-altitude asthma clinic. We conducted a pragmatic randomized controlled trial to assess the effectiveness of an internet-based self-management support app in patients who completed an in-patient pulmonary rehabilitation program at high altitude. In this trial, the primary outcome was asthma-related quality of life. In addition, we assessed asthma control and evaluated whether the degree of asthma control impacts the changes in quality of life. The process outcomes were engagement with the electronic health (eHealth) intervention and aspects of self-management and health education impact.

## Methods

### Study and Approval

The PRACTISS (Pulmonary Rehabilitation of Asthma: a Trial of sustained Internet-based Self-management Support) study is a randomized parallel group trial with 12 months of follow-up. The trial was performed from December 2012 through January 2016 in The Netherlands. The study was approved by the Medical Ethics Committee of the Leiden University Medical Center. All participants provided written informed consent upon entering the trial. The trial is registered in the Netherlands Trial Register (NTR1995) and conforms to the Helsinki Declaration.

### Patients

Patients with a diagnosis of severe asthma according to the American Thoracic Society criteria were recruited from a specialized asthma clinic. All patients completed pulmonary rehabilitation in the Dutch Asthma Centre Davos (Switzerland). They were referred to the rehabilitation clinic because previous maximal treatment did not lead to adequate asthma control. The exclusion criteria that may interfere with compliance or reliability of the measurements were relevant somatic comorbidity, serious psychological problems, and psychiatric comorbidity assessed by the Hospital Anxiety and Depression Scale [17].

### Design

The study was started during a high-altitude treatment program for severe asthma in a specialized asthma clinic. Upon enrolling in the study, approximately 1 week before completing the rehabilitation, patients were randomized via a computer-generated permuted block scheme by an independent researcher to either an internet-based strategy or usual care strategy group, with stratification for age and gender. Patients received explanations about the study procedures during the last days of their rehabilitation program, in addition to the usual preparation prior to discharge. After completing the program and finishing the baseline questionnaires, patients returned home and were treated by their own physicians who had referred them to the rehabilitation program. These physicians were notified about the participation of the patients in the study. Participants were asked to fill out questionnaires at 3, 6, 9, and 12 months.

### Intervention

Patients in the internet-based strategy group had access to various tools that supported them to achieve optimal self-management skills. For effective self-management, asthma patients need to understand the condition, the purpose of medication, and the environmental influences. Furthermore, they need to recognize worsening respiratory function and know when to seek medical attention [18]. The PatientCoach self-management support app was designed to promote attributes attained in the rehabilitation program. The self-management support modules could be customized by the health care professionals, allowing the program to be tailored to the specific needs of each patient. The modules included a written action plan, personalized asthma control questions, a daily asthma control questionnaire with lung function measurement, an actometer (Fitbit Ultra; Fitbit), patient health goals, a calendar, education provided by asthma centers, and a helpdesk. The app did not provide an option to communicate with a care provider.

The individual action plan was written by a physician from the rehabilitation clinic and included patient-tailored alarm signals, physician recommendations, and directives on when to contact health care professionals. Each patient together with the rehabilitation clinic physician formulated a patient-tailored question that served as a red flag for the patient to act upon when answered positively. In the daily asthma control questionnaire, patients could fill out the Asthma Control Questionnaire and the forced expiratory volume in one second (FEV_1_) measurement. Patients in the internet-based strategy group were provided with a small personal device (PIKO-1, NSpire Health) to measure the FEV_1_. The asthma control cutoff values that patients used to act upon were patient-tailored by the rehabilitation clinic physician. Internet support patients received an actometer (Fitbit) for daily use to measure activity. Each patient had a personal goal for daily steps. Patients formulated personal health goals together with the rehabilitation clinic physician for motivational purposes.

The calendar could be used by patients to list their health-related appointments. Patients received access to the educational material used by the rehabilitation clinic. The helpdesk was accessible for information and communication technology purposes. Several screenshots of the current version of the PatientCoach app are presented in Multimedia Appendix 1. As this was a pragmatic trial, participants were free to use the intervention as often as they liked.

### Health-Related Quality of Life

The primary outcome of this trial was health-related quality of life, which was assessed every 3 months using the validated and standardized Asthma Quality of Life Questionnaire (AQLQ) by Juniper et al [19]. A minimally clinically important difference of 0.5 was reported for the overall score [20]. The AQLQ consists of the following four subdomains: activity limitation, symptoms, emotional function, and environmental stimuli, and the overall score is determined. A higher score represents a more favorable outcome.

### Clinical Control

Every 3 months, all participants in this study were asked to fill out a questionnaire assessing clinical control (six-question Asthma Control Questionnaire [ACQ6]) as an outcome parameter for study purposes. The ACQ6 questionnaire is a validated questionnaire to measure clinical control in patients with asthma [21]. For the ACQ6, a lower score represents a more favorable outcome.

### eHealth User Engagement

User engagement was based on the amount of times a patient logged into the PatientCoach web app, as registered in the log files during the study period of 12 months.

### Health Education

The aspects of self-management and impact of health education were measured using the Health Education and Impact Questionnaire (heiQ) [22]. The heiQ is a validated questionnaire consisting of eight independent scales, providing a rich range of information about the value of patient self-management programs. The domains comprise health-directed behavior, positive and active engagement in life, negative affect, self-monitoring and insight, constructive attitudes and approaches, skill and technique acquisition, social integration and support, and health service navigation [23]. Scores range from 1 to 4, with a higher score representing favorable outcomes, except for negative affect, where a lower score represents favorable outcomes.

### Statistical Analysis

Power analysis showed that with the use of 36 patients per arm and an SD of changes in the AQLQ score of 0.75, we could expect to detect a minimally important difference of 0.5 points between AQLQ changes in the two arms (alpha=.05, power=0.80). Patients were analyzed according to the intention-to-treat approach. To correct for possible selective nonresponse, missing data were imputed using linear regression modelling. We created 100 sets of imputations using disease severity, age, gender, center, strategy, and available quality of life data as regression variables. Rubin rules were used to congregate the imputations [24]. After imputation, we constructed a linear mixed-effects model assessing the impact of the intervention on quality of life and asthma control. In this mixed model, we used strategy, follow-up, strategy × follow-up interaction, and a random intercept at the patient level to adjust for repeated measurements, as data from 3, 6, 9, and 12 months were imputed. These mixed models provided estimates for changes in asthma-related quality of life and asthma control during the follow-up period per strategy.

Patients were stratified for clinical asthma control at baseline using the ACQ6. Patients with an ACQ6 score <1.5 at baseline were considered as controlled, whereas those with an ACQ6 score ≥1.5 were considered as uncontrolled.

To define user engagement, we established a cutoff point of 12 logins per year. Patients with <12 logins were included in a low user engagement group, and those with ≥12 logins were included in a high user engagement group. We assessed whether there were differences in the AQLQ score between the user engagement groups.

To obtain insights into which self-management domains lead to improved quality of life, we used mixed models with the improvement in the AQLQ score with the internet-based strategy during follow-up as a dependent variable and the improvement in separate heiQ domains as the independent variable. All analyses were performed with Stata/IC 11.0 (Stata Corp).

## Results

### Recruitment and Baseline Characteristics

**Figure 1:**
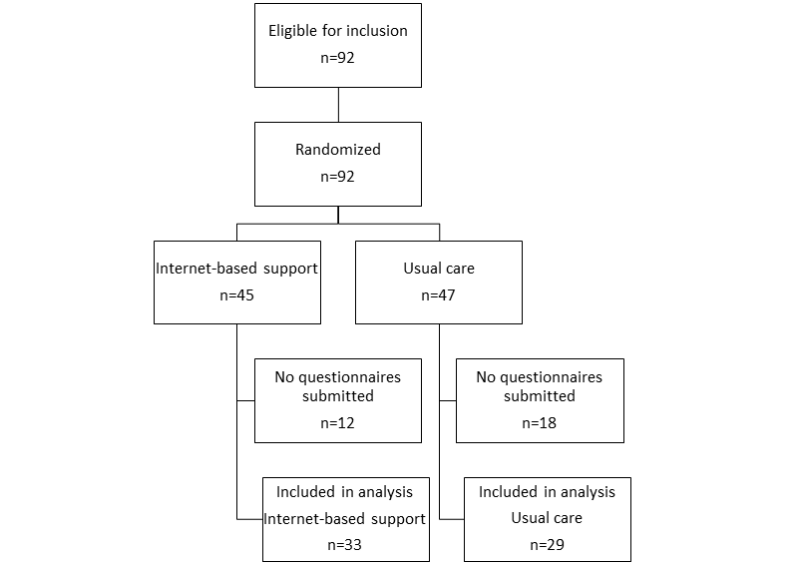
Flow diagram for the study patients.

In total, 92 patients from the Dutch Asthma Centre Davos were eligible for enrollment and were randomized after consent. However, 30 patients did not fill out the baseline questionnaire required to be included in the analysis. In our analysis, the usual care strategy group included 29 patients and the internet-based strategy group included 33 patients. Figure 1 shows the patient flow diagram. Out of 310 records (five questionnaires for 62 patients), 91 (27%) were missing, with the internet-based strategy group missing 49 questionnaires (29%) and the usual care strategy group missing 34 questionnaires (23%) (*P*=.22). All baseline questionnaires were completed.

The population characteristics at baseline are shown in Table 1. There were no relevant differences in age, sex, quality of life, and asthma between the two strategy groups. It should however be noted that clinically relevant [20], but statistically insignificant, baseline differences were found in quality of life (*P*=.11) and asthma control (*P*=.17). For both parameters, the usual care strategy group appeared to score slightly better than the internet-based strategy group. In the domain negative affect, the internet-based strategy group scored worse than the usual care strategy group.

**Table 1:** Baseline characteristics.

Characteristic	Usual care strategy (n=29), n (%) or mean (SD)	Internet-based strategy (n=33), n (%) or mean (SD)	*P* value
Age, years	44.0 (2.4)	46.7 (2.3)	.47
Male sex	9 (31%)	8 (24%)	.56
AQLQ^a^ score	5.6 (0.2)	5.2 (0.2)	.11
AQLQ-activity limitation score	5.3 (0.2)	4.8 (0.2)	.17
AQLQ-symptom score	5.7 (0.2)	5.2 (0.2)	.10
AQLQ-emotional function score	6.4 (0.1)	6.0 (0.2)	.14
AQLQ-environmental stimuli score	5.5 (0.2)	5.1 (0.2)	.31
ACQ6^b^ score	1.5 (0.2)	1.9 (0.2)	.17
heiQ^c^-behavior score	3.3 (0.1)	3.1 (0.1)	.91
heiQ-positive engagement score	3.3 (0.1)	3.3 (0.1)	.86
heiQ-negative affect score	1.4 (0.1)	1.8 (0.1)	.006
heiQ-monitoring/insight score	3.4 (0.1)	3.3 (0.1)	.52
heiQ-constructive attitude score	3.3 (0.1)	3.2 (0.1)	.53
heiQ-skill acquisition score	3.2 (0.1)	3.0 (0.1)	.06
heiQ-social support score	3.2 (0.1)	3.0 (0.1)	.07
heiQ-navigation score	3.3 (0.1)	3.3 (0.1)	.87

^a^AQLQ: Asthma Quality of Life Questionnaire.

^b^ACQ6: six-question Asthma Control Questionnaire.

^c^heiQ: Health Education and Impact Questionnaire.

### Health-Related Quality of Life

**Figure 2:**
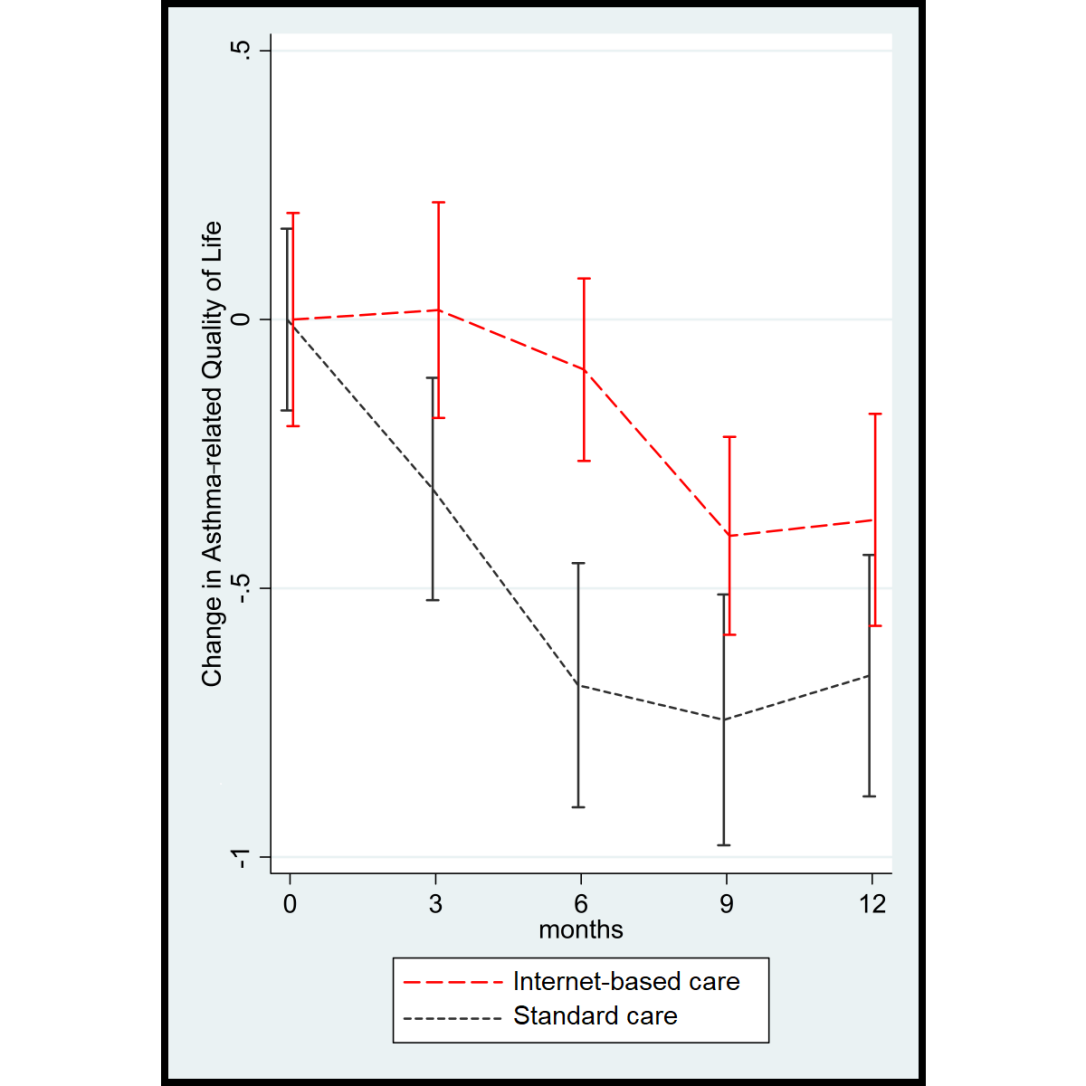
Quality of life according to the Asthma Quality of Life Questionnaire (a higher score is better) during follow-up.

The changes in asthma-related quality of life for both strategies are shown in Figure 2. Quality of life declined in both groups during follow-up, showing less decline in the internet-based strategy group. There were no relevant between-group differences in quality of life at 12 months. Analysis by a linear mixed-effects model, with treatment, strategy, follow-up × strategy interaction, and a random intercept at the patient level to adjust for repeated measurements, showed that the decline in quality of life according to the 3-month AQLQ scores (a higher AQLQ score is better) was significantly smaller during 12 months of follow-up in the internet-based strategy group than in the usual care strategy group (mean between-group AQLQ score difference 0.39, 95% CI 0.092-0.69; *P=*.01).

**Figure 3:**
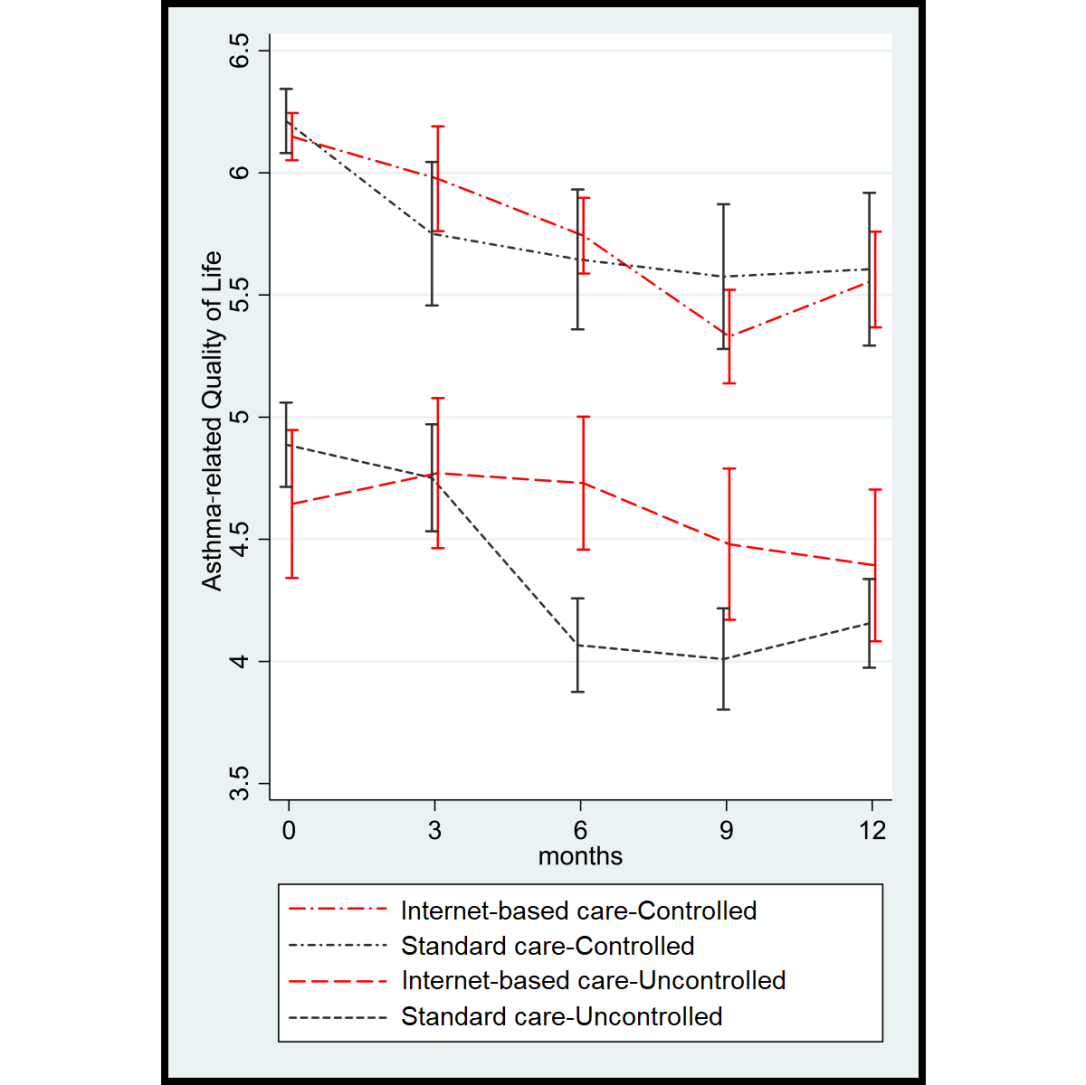
Quality of life according to the Asthma Quality of Life Questionnaire (a higher score is better) during follow-up in controlled and uncontrolled asthma patients.

A similar graph showing quality of life during follow-up using absolute values instead of changes is shown in Multimedia Appendix 2. As the baseline values for the ACQ6 (*P*=.17) and AQLQ (*P*=.11) were slightly different between the groups, but not statistically significant, we analyzed the results separately in strata according to well-established cutoff points for asthma control at baseline. This approach using stratification was also adopted in a previous study [25]. Patients with an ACQ6 score <1.5 were considered as controlled, whereas those with an ACQ6 score ≥1.5 were considered as uncontrolled. In the usual care strategy group, 16 patients were considered as controlled and 13 were considered as uncontrolled. In the internet-based strategy group, 12 patients were considered as controlled and 21 were considered as uncontrolled. Among patients with controlled asthma, a comparable decline was observed with both strategies (mean AQLQ score difference 0.07, 95% CI −0.38 to 0.53; *P*=.76) (Table 2). However, among patients with uncontrolled asthma at baseline, the decline in quality of life was relatively smaller in the internet-based strategy group than in the usual care strategy group (mean between-group AQLQ score difference 0.59, 95% CI 0.19-0.99; *P*=.003) (Figure 3).

The results of the AQLQ domains are shown in Table 3. Patients in the internet-based strategy group showed less worsening in activity limitation during follow-up as compared with the usual care strategy group (AQLQ score difference 0.44, 95% CI 0.06-0.82; *P=*.02). Overall, patients in the internet-based strategy group reported significantly less impact of asthma symptoms on quality of life during follow-up (AQLQ score difference 0.48, 95% CI 0.12-0.83; *P=*.009). Among patients with controlled asthma at baseline, no differences between the study arms were found. However, among patients with uncontrolled asthma at baseline, patients in the usual care strategy group reported significantly more impact of symptoms on quality of life, whereas those in the internet-based strategy group maintained better levels of symptom-related quality of life throughout the follow-up (AQLQ score difference 0.83, 95% CI 0.34-1.31; *P=*.001).

Similar results were found in the environmental stimuli AQLQ domain in favor of the intervention, specifically among patients with uncontrolled asthma. Overall, no relevant differences were found in the emotional functioning domain, except within the uncontrolled group, which had less impact of emotional functioning on quality of life with the internet-based strategy as compared with the usual care strategy.

**Table 2:** Asthma-related quality of life and asthma control between strategies.

Variable	Difference between baseline and follow-up, mean (95% CI)		Difference between strategies^a^, mean (95% CI)	*P* value
	Usual care strategy	Internet-based strategy		
AQLQ^b^	−0.60 (−0.82 to −0.38)	−0.21 (−0.42 to −0.01)^c^	0.39 (0.092 to 0.69)^c^	.01
AQLQ, controlled patients	−0.57 (−0.87 to −0.27)	−0.50 (−0.84 to −0.15)^c^	0.07 (−0.38 to 0.53)	.76
AQLQ, uncontrolled patients	−0.64 (−0.95 to −0.33)	−0.05 (−0.30 to 0.19)	0.59 (0.19 to 0.99)^c^	.003
ACQ6^d^	0.67 (0.41 to 0.93)	0.17 (−0.08 to 0.41)	−0.50 (−0.86 to −0.15)^c^	.006
ACQ6, controlled patients	0.74 (0.37 to 1.11)^c^	0.70 (0.27 to 1.13)^c^	−0.038 (−0.60 to 0.53)	.89
ACQ6, uncontrolled patients	0.59 (0.24 to 0.94)^c^	−0.14 (−0.41 to 0.14)	−0.73 (−1.18 to −0.28)^c^	.002

^a^An AQLQ score difference >0 and an ACQ6 score difference <0 reflect a positive effect of the intervention.

^b^AQLQ: Asthma Quality of Life Questionnaire.

^c^Statistically significant.

^d^ACQ6: six-question Asthma Control Questionnaire.

**Table 3:** Asthma-related quality of life subdomains between strategies.

Variable	Difference between baseline and follow-up, mean (95% CI)			Difference between strategies, mean (95% CI)		*P* value
	Usual care strategy	Internet-based strategy				
AQLQ^a^-activity limitation	−0.73 (−1.00 to −0.45)^b^	−0.29 (−0.54 to −0.03)^b^	0.44 (0.06 to 0.82)^b^		.02	
AQLQ-activity limitation, controlled patients	−0.86 (−1.26 to −0.46)^b^	−0.50 (−0.96 to −0.03)^b^	0.36 (−0.25 to 0.97)		.24	
AQLQ-activity limitation, uncontrolled patients	−0.57 (−0.95 to −0.18)^b^	−0.17 (−0.47 to 0.14)	0.40 (−0.09 to 0.89)		.11	
AQLQ-symptoms	−0.65 (−0.91 to −0.39)^b^	−0.17 (−0.41 to 0.07)	0.48 (0.12 to 0.83)^b^		.009	
AQLQ-symptoms, controlled patients	−0.56 (−0.91 to −0.22)^b^	−0.61 (−1.00 to 0.21)	−0.04 (−0.57 to 0.48)		.88	
AQLQ-symptoms, uncontrolled patients	−0.75 (−1.13 to −0.37)^b^	0.08 (−0.22 to 0.38)	0.83 (0.34 to 1.31)^b^		.001	
AQLQ-environmental stimuli	−0.63 (−0.86 to −0.40)^b^	−0.32 (−0.53 to −0.10)^b^	0.32 (0.01 to 0.63)^b^		.047	
AQLQ-environmental stimuli, controlled patients	−0.54 (−0.84 to −0.24)^b^	−0.42 (−0.76 to −0.07)^b^	0.12 (−0.34 to 0.58)		.60	
AQLQ-environmental stimuli, uncontrolled patients	−0.75 (−1.10 to −0.41)^b^	−0.26 (−0.53 to 0.01)	0.49 (0.05 to 0.93)^b^		.028	
AQLQ-emotional function	−0.19 (−0.40 to 0.02)	−0.07 (−0.27 to 0.13)	0.12 (−0.17 to 0.41)		.43	
AQLQ-emotional function, controlled patients	0.04 (−0.22 to 0.29)	−0.30 (0.60 to 0.00)	−0.33 (−0.73 to 0.06)		.10	
AQLQ-emotional function, uncontrolled patients	−0.46 (−0.79 to 0.14)	0.06 (−0.20 to 0.32)	0.52 (0.10 to 0.94)^b^		.02	

^a^AQLQ: Asthma Quality of Life Questionnaire.

^b^Statistically significant.

### Asthma Control

**Figure 4:**
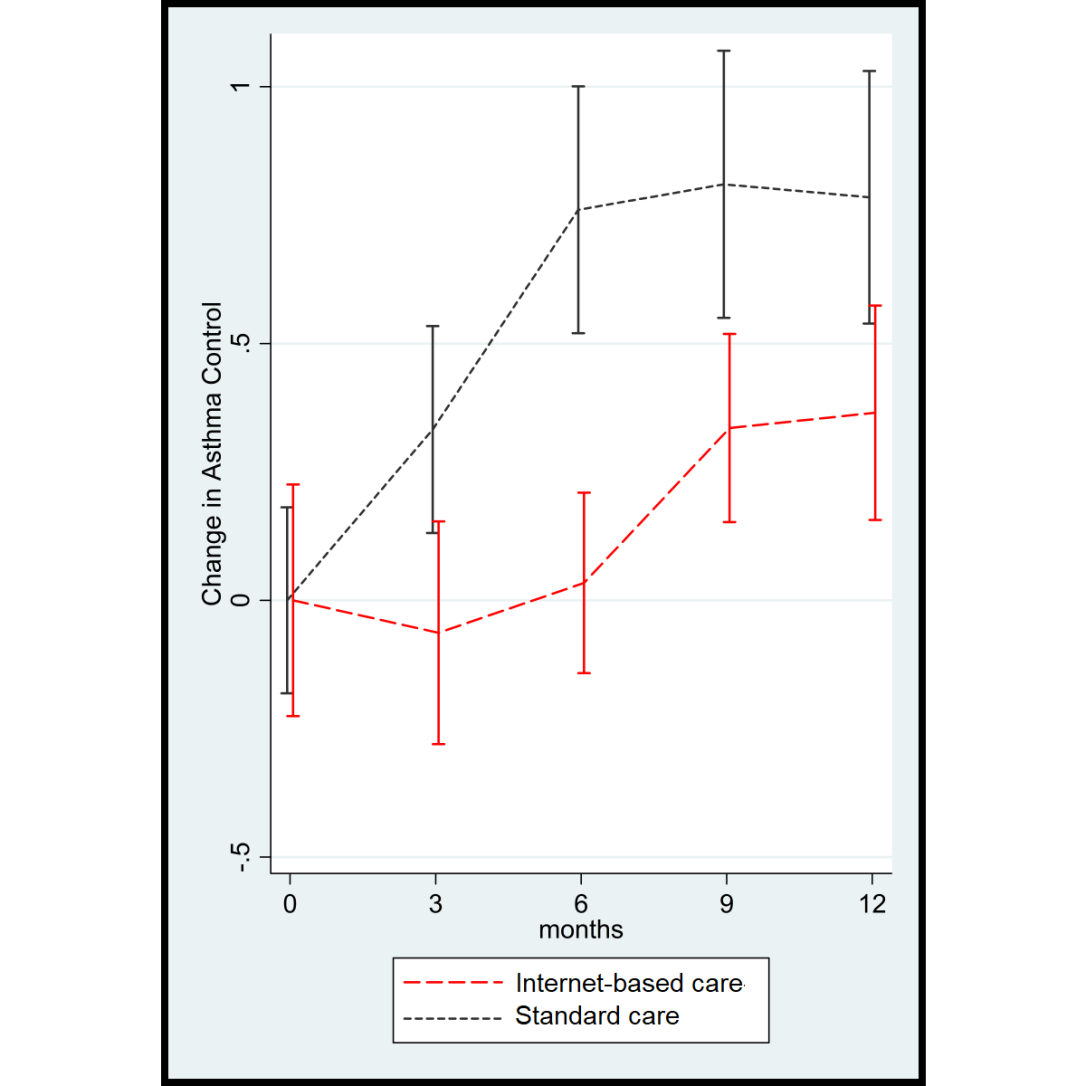
Asthma control according to the Asthma Control Questionnaire (a lower score is better) during follow-up.

The favorable findings in quality of life for the internet-based strategy were confirmed by changes in asthma control. A similar mixed model showed a favorable and statistically significant change in asthma control (a lower ACQ6 score is better) in the internet-based strategy group during follow-up as compared with the usual care strategy group (mean ACQ6 score difference −0.50, 95% CI −0.86 to −0.15; *P=*.006) (Figure 4).

**Figure 5:**
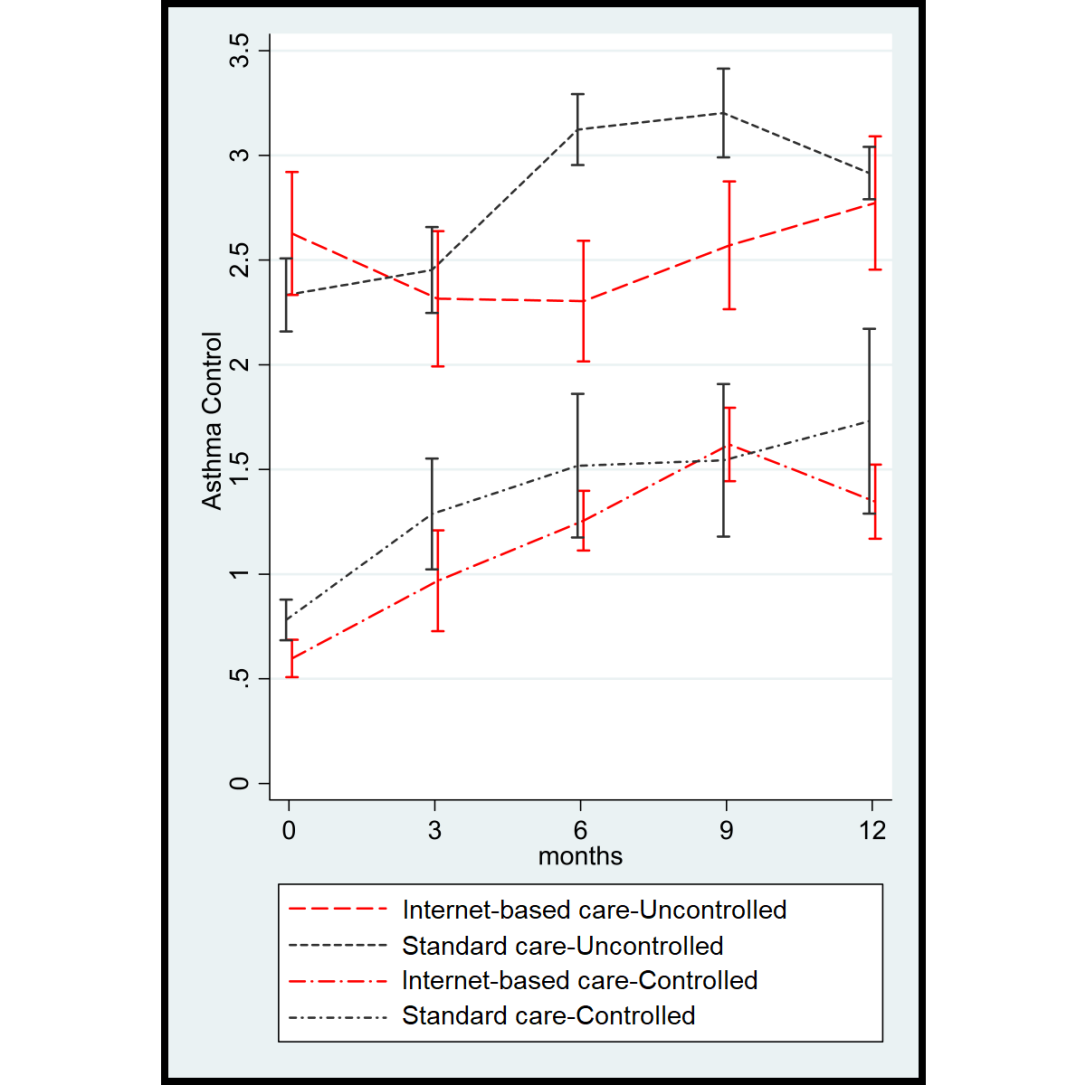
Asthma control according to the Asthma Control Questionnaire (a lower score is better) during follow-up in controlled and uncontrolled asthma patients.

A similar graph showing asthma control during follow-up using absolute values instead of changes is shown in Multimedia Appendix 3. With respect to the stratified analysis, a similar pattern was observed for asthma control. Among controlled patients, both groups showed a comparable trend of declining asthma control during follow-up (mean ACQ6 score difference 0.04, 95% CI −0.60 to 0.53, *P=*.89) (Figure 5). However, among uncontrolled patients, a clinically and significantly better asthma control level was sustained with the internet-based strategy (mean ACQ score difference −0.73, 95% CI −1.176 to −0.277; *P=*.002), and this was most noticeable at 6 and 9 months (Figure 5).

### eHealth User Engagement

**Figure 6:**
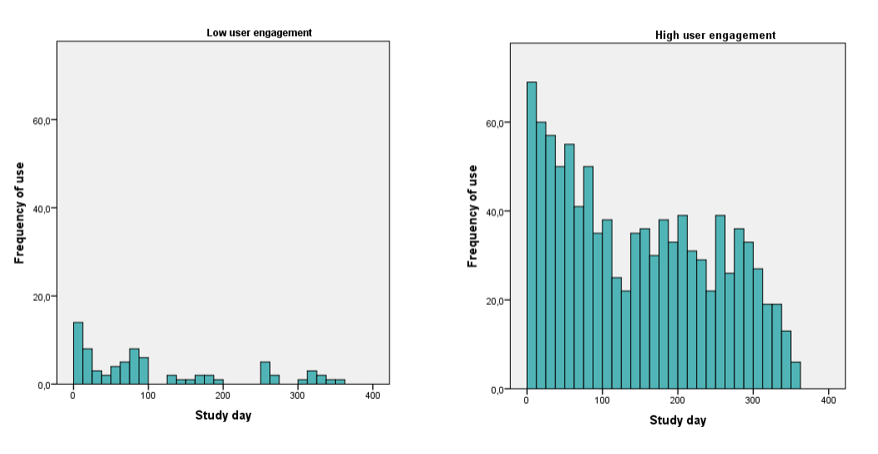
Login frequency in low (left panel; n=18) and high (right panel; n=13) user engagement groups.

**Figure 7:**
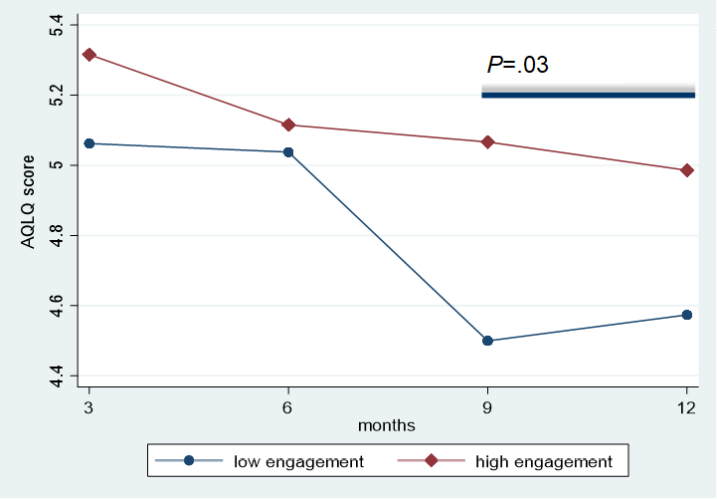
Asthma-related quality of life in low and high user engagement groups. AQLQ: Asthma Quality of Life Questionnaire.

The results with respect to eHealth user engagement are depicted in Figures 6 and 7. There was a decline in login frequency in both the low and high user engagement groups. During the last 6 months of follow-up, asthma-related quality of life was significantly better maintained in the high user engagement group than in the low user engagement group (mean AQLQ score difference −0.39, 95% CI 0.049-0.73; *P=*.03).

### Health Education Impact

The results of the mixed models for health education impact are shown in Table 4. We assessed whether the improvement in asthma-related quality of life could be explained by the changes in the separate heiQ domains. Improvement in the domain of health care navigation during follow-up was positively related to improvement in quality of life (AQLQ score difference 0.55, 95% CI 0.06-1.04; *P=*.03) in patients with access to the intervention. Although the domains of health-directed behavior, positive engagement, and monitoring and insight suggested a positive relationship with asthma-related quality of life (effect estimates of 0.28 [*P*=.19], 0.29 [*P*=.19], and 0.40 [*P*=.18] points, respectively), none of these domains reached statistical significance.

**Table 4:** Relation between improvement in health education impact domains and improvement in quality of life.

heiQ^a^ domains	Mixed model effect sizes^b^ (β), mean (95% CI)	*P* value
Health-directed behavior	0.28 (−0.14 to 0.70)	.19
Positive engagement	0.29 (−0.15 to 0.73)	.19
Negative affect	−0.20 (−0.60 to 0.19)	.31
Monitoring and insight	0.40 (−0.18 to 0.97)	.18
Constructive attitude	0.15 (−0.26 to 0.57)	.47
Skill acquisition	0.03 (−0.58 to 0.64)	.92
Social support	0.13 (−0.24 to 0.49)	.49
Health care navigation	0.55 (0.06 to 1.04)	.03

^a^heiQ: Health Education and Impact Questionnaire.

^b^In the domain of negative affect, a negative effect size reflects a favorable outcome, whereas in the other domains, a positive effect size reflects favorable outcomes.

## Discussion

In this study, we assessed the effectiveness of internet-based self-management support in addition to usual care in patients with severe asthma after completing high-altitude climate treatment. Both the internet-based strategy and usual care strategy groups showed declines in asthma-related quality of life and asthma control during follow-up. This trend was expected as patients were enrolled upon completing a pulmonary rehabilitation just before returning to their home situation at sea level. However, the declines in both endpoints in the internet-based strategy group were significantly smaller than those in the usual care strategy group. In particular, in the internet-based strategy group, patients who still exhibited uncontrolled asthma at discharge did not show a further decline in quality of life in contrast to their counterparts in the usual care strategy group.

The individual domains of the AQLQ (activity limitation, symptoms, emotional function, and environmental stimuli) showed results similar to those of the AQLQ aggregated score described above. The most prominent results were found within the symptoms domain, where patients with controlled asthma reported an increase in symptoms during follow-up, with comparable effect sizes between the study arms. However, among patients with uncontrolled asthma at baseline, those who received the usual care strategy reported clinically relevant increases in symptoms, whereas those who received the internet-based strategy reported no increase in symptoms.

Asthma control did not decline greatly during follow-up in the internet-based strategy group, whereas asthma control declined in the usual care strategy group. Similar to the findings for quality of life, no significant difference in the decline in asthma control was found between the study groups among patients with controlled asthma at discharge. However, among patients with uncontrolled asthma, no decline in asthma control was observed with the internet-based strategy.

There are sparse data regarding the effectiveness of self-management support strategies in patients with severe asthma after completion of high-altitude climate treatment. Our results are consistent with the findings of previously published articles regarding interventions in patients with mild asthma [10-12,15]. In addition, our results substantiate the findings of a recently published uncontrolled observational study reporting sustained improvements in asthma control and quality of life after high-altitude climate treatment in the years before this study started [6,7]. To our knowledge, this is the first study to assess the effectiveness of internet-based self-management support in patients with severe asthma after completing high-altitude climate treatment.

Several possible limitations in this study need to be addressed, including possible selection bias, the nonresponse rate, and statistical power. As this was a self-management study and a pragmatic trial, we did not spend large amounts of time in motivating people during follow-up to fill out questionnaires, as this would be an intervention by itself and was not possible in the clinical setting after implementation. Therefore, in our data set, 27% (91/310) of possible entries were missing. However, as the rates of missing data in both groups were comparable, we assumed that there was no impact on the internal validity of the study. In addition, the relatively high drop-out rate is consistent with an earlier study in patients with severe asthma [6]. It is not clear whether this is due to omissions within the studies or the requirement of an alternative approach for the population to adhere to the study protocol. Another explanation might involve patients’ initial willingness to cooperate with a study conducted by the rehabilitation center where they stayed for many weeks, resulting in the inclusion of patients without a high intrinsic motivation, who are usually not included in a typical 12-month trial. We imputed missing data using linear regression modelling with 100 sets of imputations, thereby minimizing the impact of missing data.

As we calculated the differences between baseline and follow-up, it could be argued that our results might be explained by selection bias as the usual care population had better, although not statistically significant, asthma-related quality of life and asthma control at baseline. Therefore, there might be more room for a decline in the follow-up period. To correct for this possible bias, we stratified for asthma control at baseline, showing that quality of life and asthma control did not decrease in patients with uncontrolled asthma. We therefore conclude that this bias does not explain the current results.

Not all differences found in this study reached statistical significance, possibly due to the limited number of patients enrolled. Based on our power analysis, we aimed to enroll 72 patients (36 patients per arm) to have sufficient power for the detection of a minimally important difference. Of the 92 eligible patients, 62 contributed to the study. However, we had sufficient power to detect a minimally important and consistent difference of 0.5 points in the AQLQ and ACQ. Therefore, despite the fact that the number of patients could still be regarded as a limitation, we feel that the number of patients included was sufficient for our conclusions.

A further limitation of this study is that the results were based upon self-reported outcome measures, as we had no access to FEV_1_ measurements or other biomarkers. Although such markers would definitely be of additional value, we feel that by using well-validated instruments our self-reported outcomes are sufficiently robust to draw conclusions.

One of the strengths of this study is the pragmatic approach we adopted. Patients were included by members of the rehabilitation center, indicating the feasibility of imbedding a self-management program on completing the rehabilitation program. The patients had access to a helpdesk service for technical support, but no follow-up from the rehabilitation center or the patient’s own physician was required for support during the intervention. Certainly, regular visits to their own physicians continued to be applicable. Although this study does not amount to a formal feasibility study, the usability, accessibility, and more than monthly use of the intervention in half of the intervention group suggest that this self-management support intervention might be feasible without investments other than the introduction to the program during follow-up and the costs for technical maintenance of the system.

These findings confirm the presumption that patients with severe asthma who complete a high-altitude climate treatment deteriorate at a group level in terms of asthma control and quality of life after discharge. Partially, this could be attributed to regression to the mean, as patients leave the rehabilitation in an optimized health status. However, the results suggest that this decline could be reduced by offering an internet-based self-management program specifically in patients who leave rehabilitation with uncontrolled asthma (ACQ score ≥1.5). These patients represent the group of patients in which achieving asthma control is most complicated. Additionally, without self-management support intervention, their quality of life and asthma control declined even further, resulting in lower scores than patients who were able to achieve asthma control during rehabilitation. However, with an internet-based self-management program, this group may be able to stabilize their symptoms, which is an important and clinically relevant improvement.

In this study, the results were achieved without any interference by health care professionals in the internet-based self-management program after discharge, suggesting that the intervention is easily feasible for implementation and the approach might be very cost-effective, although this needs to be confirmed in future research.

Other patients with asthma who have not completed rehabilitation might also benefit from such interventions. For instance, patients discharged after asthma-related hospital admission may benefit from the same self-management support tools. The support tools are easily tailored to patients with various degrees of severity, enabling health care providers to provide this intervention to patients with less severe asthma. Access to the internet has become ever more widespread, implying that internet-based self-management tools are already accessible to most patients in developed countries. The large dropout rate emphasizes the importance of motivating people prior to enrollment in postrehabilitation self-management interventions. Future research is needed as evidence of self-management support in patients with severe asthma remains limited. The assessment of lung function and biomarkers may be of additional value to establish the effectiveness of self-management outcomes.

In conclusion, internet-based self-management support is an effective addition to usual care in patients with severe asthma on completion of a pulmonary rehabilitation program. Implementation of such interventions is feasible in clinical practice and may contribute to the stabilization of symptoms in patients with severe asthma.

## Appendix

Multimedia Appendix 1 Screenshots of the mobile version of the PatientCoach app.

Multimedia Appendix 2 Quality of life in absolute values (a higher score is better) during follow-up.

Multimedia Appendix 3 Asthma control in absolute values (a lower score is better) during follow-up.

Multimedia Appendix 4 CONSORT-eHEALTH checklist (V 1.6.1).

## Abbreviations

ACQ: Asthma Control Questionnaire
ACQ6: six-question Asthma Control Questionnaire
AQLQ: Asthma Quality of Life Questionnaire
eHealth: electronic health
FEV1: forced expiratory volume in one second
heiQ: Health Education and Impact Questionnaire
