# Supplementary material for: Internet-Based Self-Management Support After High-Altitude Climate Treatment for Severe Asthma: Randomized Controlled Trial
Source: J Med Internet Res. 2020 Jul 22;22(7):e13145. doi: 10.2196/13145 (PMC7407281; doi:10.2196/13145)

Screenshot 1. Graphs of personalized question related to asthma control and lung function.

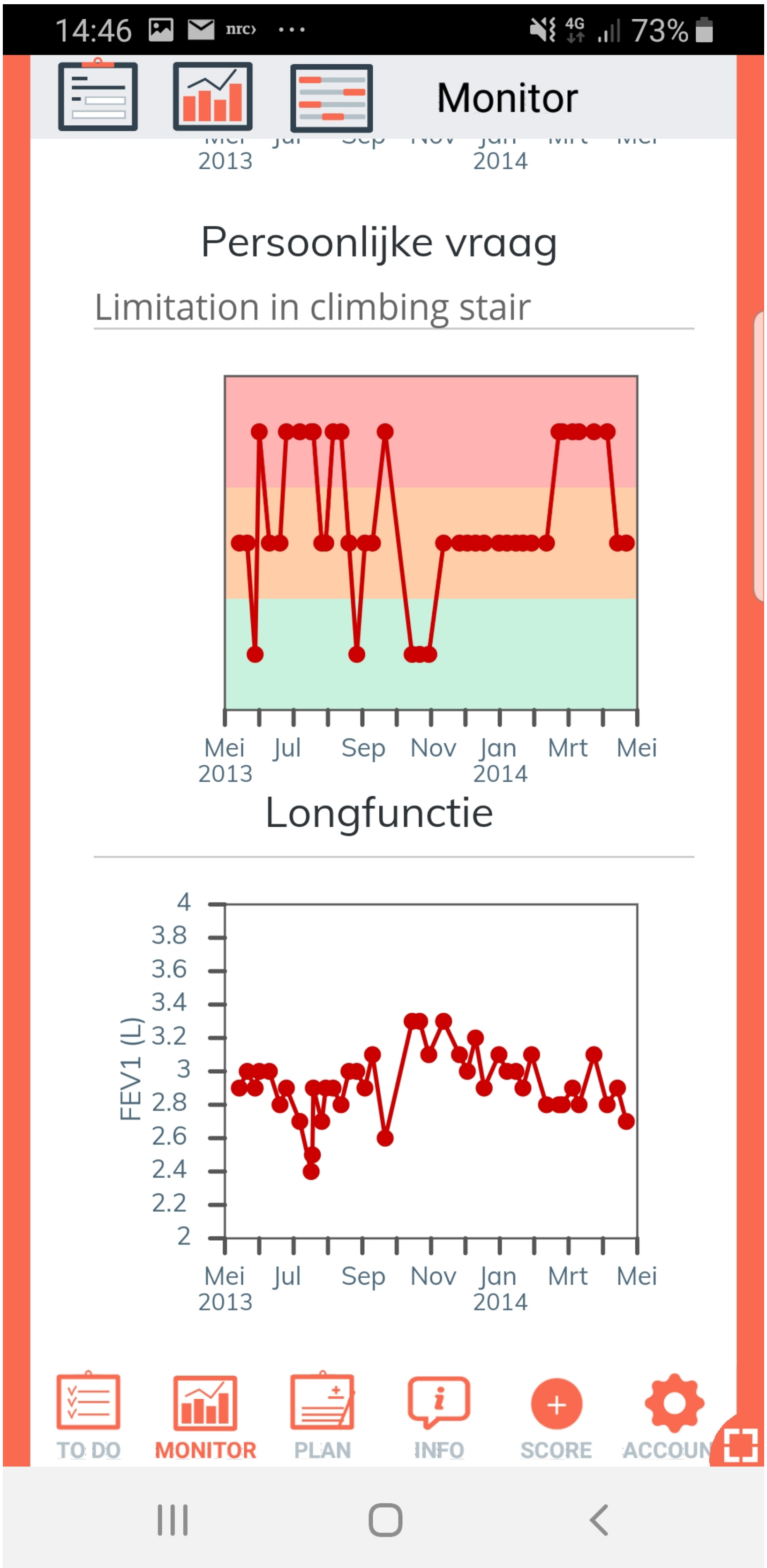

Screenshot 2. Links to education materials.

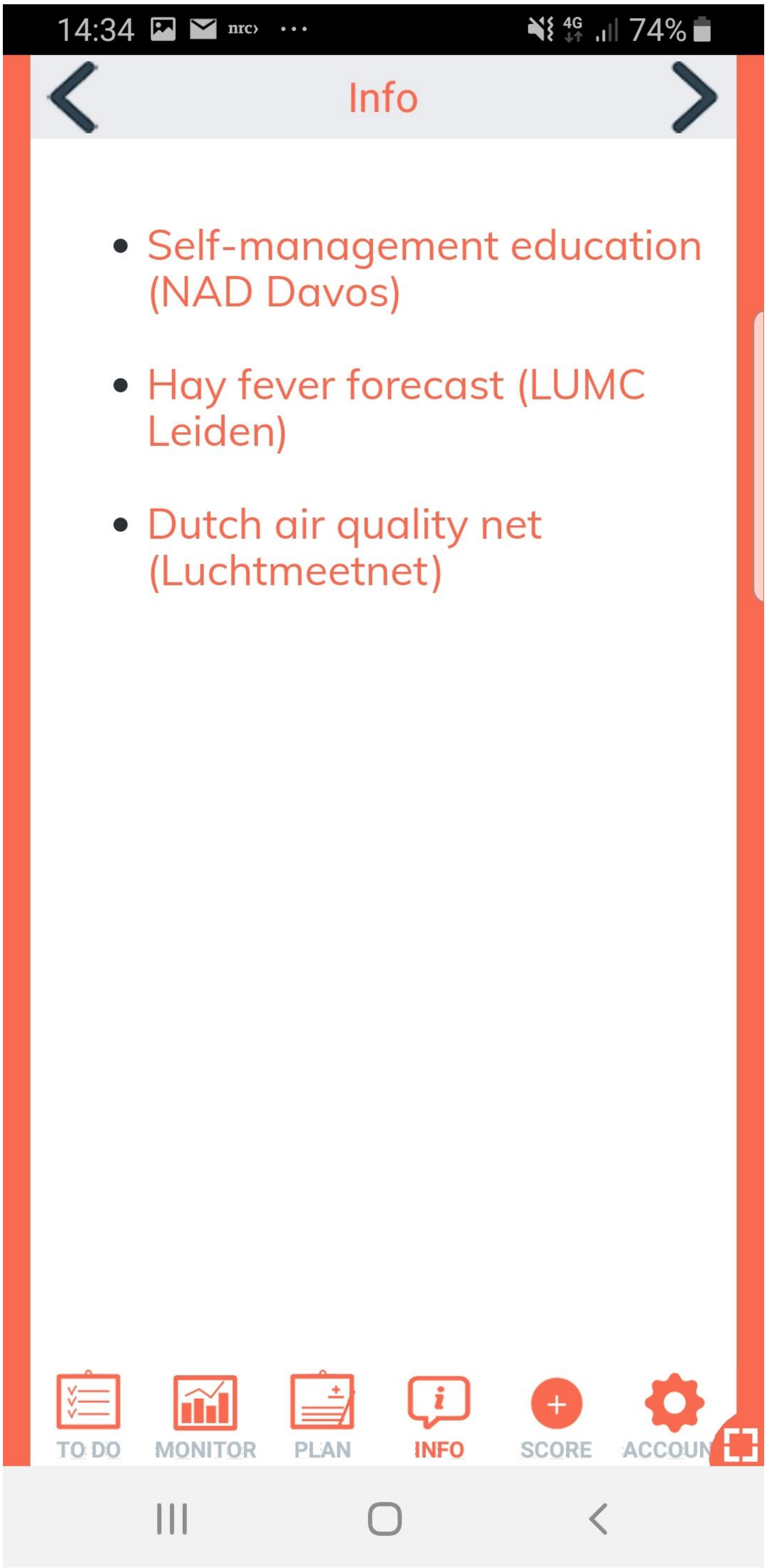

Screenshot 3. Graphs of daily symptom score and actometer (steps per day).

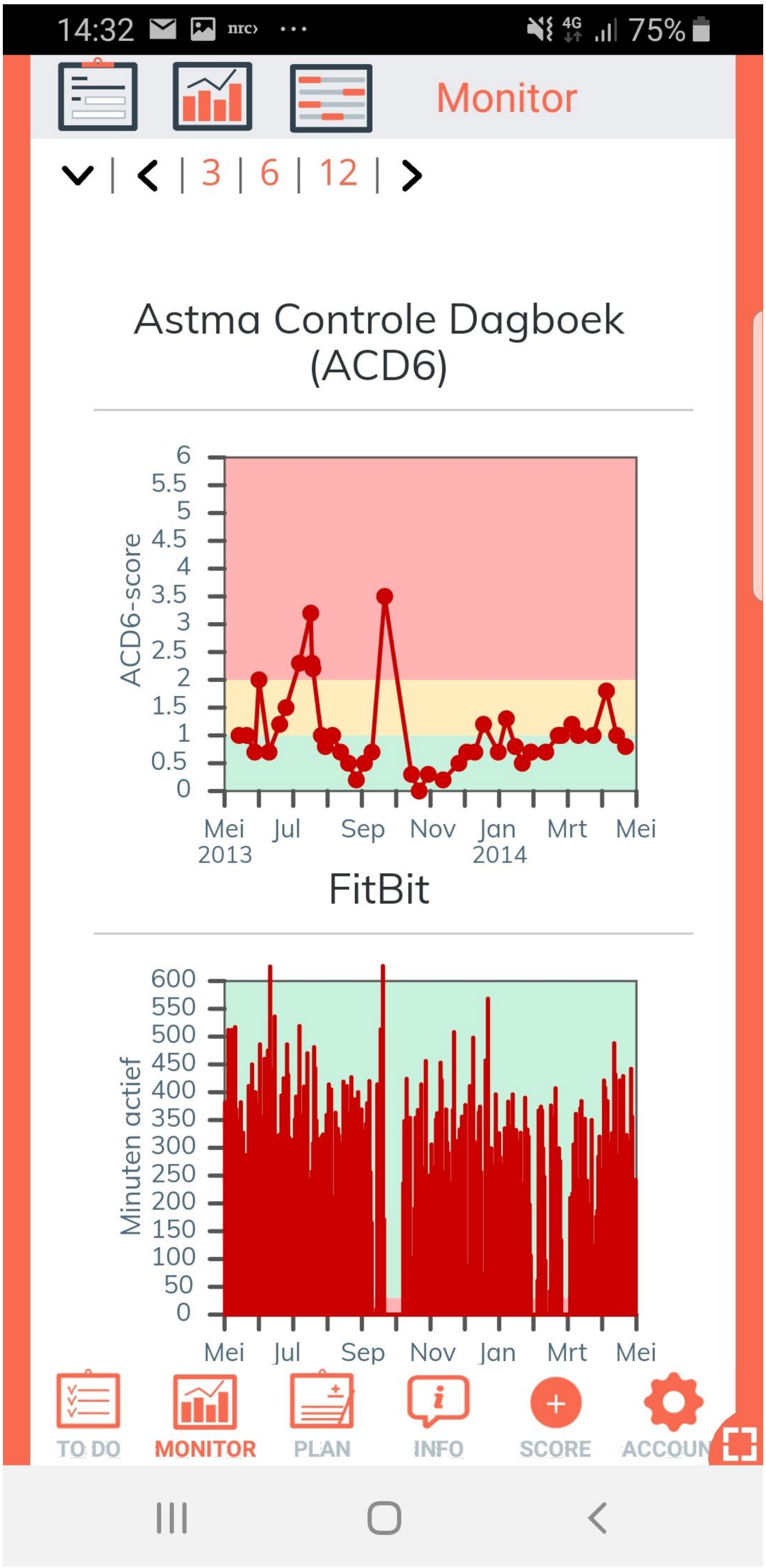

Screenshot 4. Personalized goal and action plan.

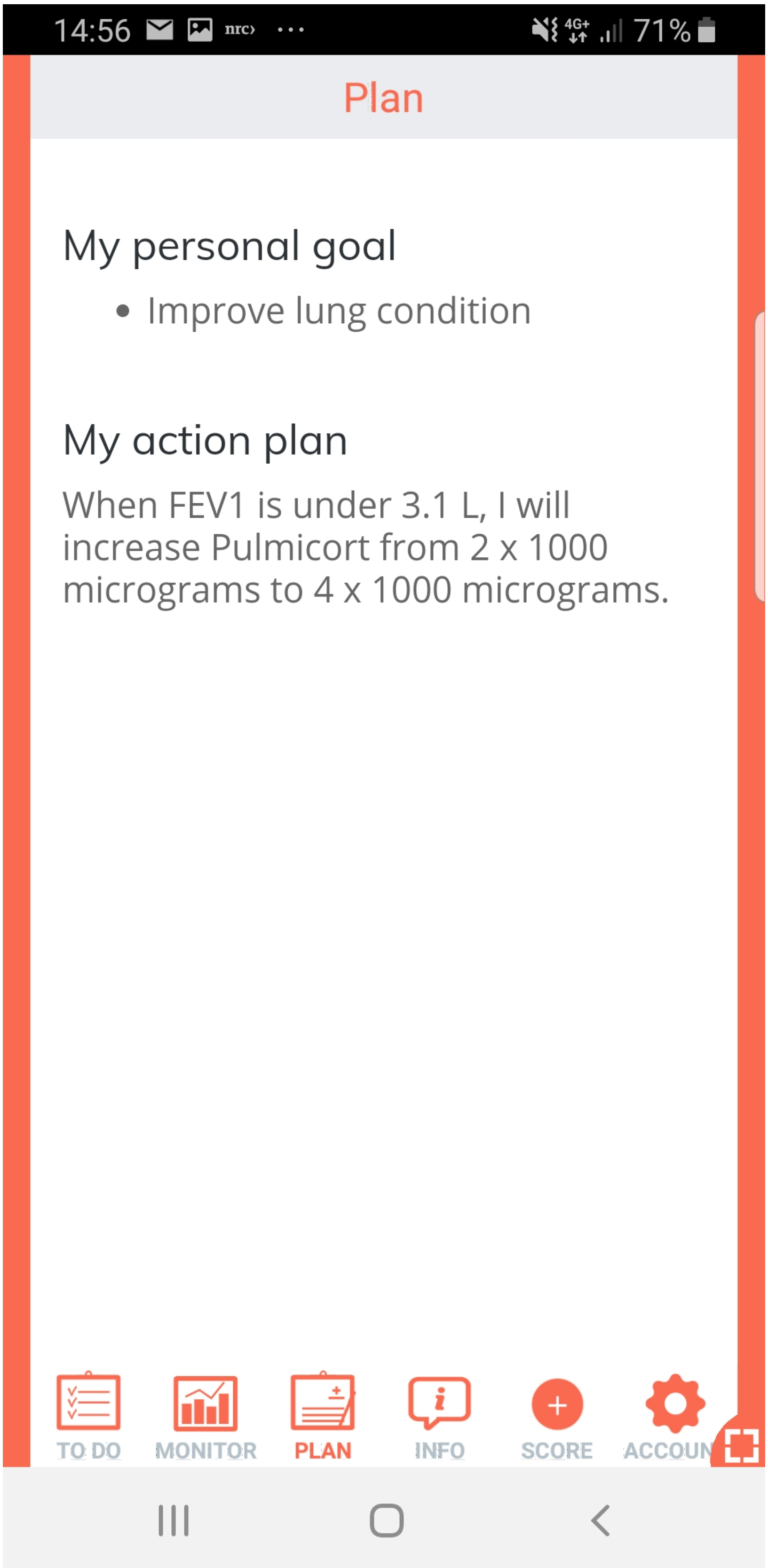

Supplement: Multimedia Appendix 1 [file jmir_v22i7e13145_app1.pdf]
